# Chronic Inflammatory Disease and Osteopathy: A Systematic Review

**DOI:** 10.1371/journal.pone.0121327

**Published:** 2015-03-17

**Authors:** Luca Cicchitti, Marta Martelli, Francesco Cerritelli

**Affiliations:** 1 Clinical-based Human Research Department, C.O.ME. Collaboration, Pescara, Italy; 2 Research Department, Accademia Italiana Osteopatia Tradizionale, Pescara, Italy; 3 Department of Neuroscience, Imaging and Clinical Sciences “G. D'Annunzio” University of Chieti-Pescara; ITAB-Institute for Advanced Biomedical Technologies, “G. D'Annunzio” University of Chieti-Pescara, Italy; Queen Mary University of London, UNITED KINGDOM

## Abstract

**Background:**

Chronic inflammatory diseases (CID) are globally highly prevalent and characterized by severe pathological medical conditions. Several trials were conducted aiming at measuring the effects of manipulative therapies on patients affected by CID. The purpose of this review was to explore the extent to which osteopathic manipulative treatment (OMT) can be benefi-cial in medical conditions also classified as CID.

**Methods:**

This review included any type of experimental study which enrolled sub-jects with CID comparing OMT with any type of control procedure. The search was conducted on eight databases in January 2014 using a pragmatic literature search approach. Two independent re-viewers conducted study selection and data extraction for each study. The risk of bias was evaluated according to the Cochrane methods. Heterogeneity was assessed and meta-analysis performed where possible.

**Results:**

10 studies met the inclusion criteria for this review enrolling 386 subjects. The search identified six RCTs, one laboratory study, one cross-over pilot studies, one observation-al study and one case control pilot study. Results suggest a potential effect of osteopathic medicine on patients with medical pathologies associated with CID (in particular Chronic Obstructive Pul-monary Disease (COPD), Irritable Bowel Syndrome, Asthma and Peripheral Arterial Disease) com-pared to no treatment or sham therapy although data remain elusive. Moreover one study showed possible effects on arthritis rat model. Meta-analysis was performed for COPD studies only show-ing no effect of any type of OMT applied versus control. No major side effects were reported by those receiving OMT.

**Conclusion:**

The present systematic review showed inconsistent data on the effect of OMT in the treatment of medical conditions potentially associated with CID, however the OMT appears to be a safe approach. Further more robust trials are needed to determine the direction and magnitude of the effect of OMT and to generalize favorable results.

## Introduction

Chronic inflammatory disease (CID) is a medical condition characterized by chronic inflammation, defined as a prolonged and persistent pro-inflammatory state marked chiefly by new connective tissue formation [1]. A number of diseases are included into this category such as autoimmune diseases, metabolic syndrome, neurodegenerative diseases, chronic obstructive pulmonary disease (COPD), chronic inflammatory bowel disease, cardiovascular diseases.

The number of people suffering from CIDs has been increasing over the last three decades. An epidemiological screening documented that CIDs are the largest cause of death in the world. In 2002, the leading chronic diseases (cardiovascular disease, chronic respiratory disease and diabetes) caused 29 million deaths [2].

Worldwide the annual mortality due to CID is expected to increase. By 2030, it has been estimated that 171 million people will be affected by CID in the United States [3]. A number of factors are recognized as causes of the rise: misuse of antibiotics, vitamin supplementation, war, and overuse of immune-modulatory or immunosuppressive agents.

Several research were conducted to identify pathogenetic mechanisms behind CID [4–7]. Three theories are currently under consideration: 1) reaction to a persistent antigen, that can be also represented by environmental factors such as smoke or foods [8]; 2) genetic components, with multiple genes possibly involved [9]; 3) inappropriate host immune response to ubiquitous antigens [7]. In some cases the genesis of the pathology can be multifactorial.

Current treatment for CID requires a long-lasting use of anti-inflammatory drugs (steroid and/or non-steroid) that, in some cases could produce severe side effects [8]. A recent review highlighted that current medications used to treat CID suppress the symptoms but prevent the complete resolution of the disease, leading to a persistent low-grade inflammation. Authors claimed that chronic use of anti-inflammatory medication could impede the body from making a full recovery [8].

Only one review has published on the prevalence of the use of complementary and alternative medicines (CAM) in people affected by pathologies associated with CID [10]. Clinical studies suggested that CAM could have a role in improving CID symptoms but results remain elusive [11, 12].

Considering osteopathy, no reviews were published looking at estimating the effect of osteopathic manipulative treatment (OMT) in patients with medical conditions also classified as CID. OMT is a drug-free manual medicine which uses manipulations to treat somatic dysfunctions (ICD-10 code: M99.0–99.9). Several studies demonstrated the anti-inflammatory effect of OMT both in vitro and in vivo [13–16]. Bench research showed that the use of mechanical strain patterns mimicking the osteopathic techniques produced a reduction of IL-6, 12, substance P and TNFa [13, 14, 16]. Clinical studies on humans demonstrated the anti-inflammatory effect of OMT reducing several cytokines, including substance P [15]. In addition, the tuning on the autonomic nervous system has been suggested as another mechanism by which OMT can act. This hypothesis is based on the increase of parasympathethic activity leading to a trophotropic effect of OMT [17]. Therefore, the objective of this systematic review was to provide an overview on the use and effect of OMT as alternative therapy in treating pathologies associated with CID.

## Materials and Methods

### Criteria for considering studies for this review

#### Types of studies

This research included single- and multi- center randomized controlled trial (RCT), *quasi*-RCT and controlled clinical trials. Interrupted time series (ITS) studies, controlled before and after (CBA) studies, observational studies, cohort studies, cross-sectional studies, case-control, case-series and case-report studies were also included because of the lack of controlled studies on the topic. Study reports must have been written in English. Abstracts were also excluded.

#### Types of participants

This review included subjects with medical conditions classified also as CID, of either gender and any age. Moreover, studies with animal models were included in order to provide a wide variety of casuistry.

#### Types of interventions

Included studies had to assess the effect of OMT compared with one or more of the following control groups: no treatment, placebo/sham, usual/routine care, or waiting list control. Interventions could be applied alone or in addition to conventional treatments (i.e. pharmacological co-interventions, counseling or advice prescription).

#### Types of outcome measures

The primary outcome for this review was to quantify the effectiveness of OMT in patients with medical pathologies associated with CID compared with any other type of complementary medicine or usual medical care.

Secondary outcome was to analyze data on side effects caused by osteopathic treatment.

#### Search methods for identification of studies

The identification of the studies was conducted by a comprehensive computerized search of MEDLINE (http://www.ncbi.nlm.nih.gov/pubmed), Scholar google (http://scholar.google.it), SCOPUS (http://www.elsevier.com/online-tools/scopus), clinicaltrial.gov, chiloras/MANTIS, OSTMED.DR (http://ostmed-dr.com/), Osteopathic Research Web (http://www.osteopathic-research.com/) and the Cochrane Library (http://www.thecochranelibrary.com). Other sources were considered as follows: grey literature, national trials registers, web searching, conference proceedings. Search terms included: osteopathic manipulative treatment(Free Terms), chronic inflammatory disease(MeSH Terms), bronchitis(MeSH Terms), pulmonary disease, chronic obstructive(MeSH Terms), asthma(MeSH Terms), pelvic inflammatory disease(MeSH Terms), prostatitis(MeSH Terms), otitis(MeSH Terms), vestibular neurotides(MeSH Terms), middle ear inflammation(MeSH Terms), neuropathy(MeSH Terms), polyradiculoneuropathy, chronic inflammatory demyelinating(MeSH Terms), myelitides(MeSH Terms), brain inflammation(MeSH Terms), inflammatory bowel disease(MeSH Terms), irritable bowel syndromes(Free Terms), gallbladder inflammation(Free Terms), gastritides(MeSH Terms), atherosclerosis(MeSH Terms), vasculitides(MeSH Terms), mediastinum inflammation(Free Terms), cardiomyopathy, arteritides, bone inflammation(Free Terms), arthritides(MeSH Terms), rheumatic disease(Free Terms). The research was conducted from journal inception to January 2014.

Duplicate records were identified in EndNOTE and eliminated.

### Data collection and analysis

#### Selection of study

The two reviewers (LC and MM) with expertise in osteopathic medicine conducted independently study selection based on the explicit search strategy. Discrepancies were resolved by consensus with FC as an arbiter. There were discussions about two studies that in the end were excluded.

According to inclusion criteria, reviewers independently screened titles and abstracts and full-text were retrieved and assessed.

#### Data extraction and management

Data extraction was performed independently by the two reviewers, in terms of number of patients, type of interventions, study results and all the other descriptive characteristics of the included trials. All disagreements were discussed and reported by consensus. If data was not reported in the study, the author was contacted. All the analyzed data was stored in a dedicated hard disk, accessible only by the two reviewers.

#### Assessment of risk of bias in included studies

Each study was, independently, evaluated by the two reviewer authors. According to the Cochrane methods, the risk of bias was categorized in low, high and unclear across the following domains: Sequence Generation; Allocation Concealment; Blinding to Personnel; Blinding to Outcome Analysis and other bias [18]. Tools proposed by the Cochrane Effective Practice and Organization of Care Group were used to evaluate the risk of bias for CBA and ITS studies [19].

#### Measures of treatment effect

For continuous data, mean differences with 95% confidence intervals (MD; 95% CI) were used. For dichotomous outcomes, results were presented as relative risk (RR) with 95% confidence intervals (CI).

#### Dealing with missing data

With regard to missing data, the authors were contacted for more information also to know the reason for the absence. Where missing data were known the reasons were described.

#### Assessment of heterogeneity

Studies were not pooled if there was significant heterogeneity. This may be methodological, statistical or clinical heterogeneity. Heterogeneity was assessed using the I^2^ statistic, which assessed how much of the variation between studies is due to heterogeneity rather than to chance [20]. Values over 50% suggested substantial heterogeneity, and values over 75% suggested considerable heterogeneity, but its significance also depended upon the magnitude and direction of the effect, and the strength of the evidence, e.g. the p value from a statistical test. Funnel plots were used to help identify possible publication bias [20].

#### Data synthesis

An intention-to-treat analysis i.e. including all those randomized to their original groups, whether or not they remained in the study, was conducted. Data was reported as mean, point estimate, percentage and range. Dispersion was presented as standard deviation (SD) and 95% confidence interval (CI). The relative risk (RR) was greater than 1 if more patients were successfully treated by the osteopathy group compared with the intervention group. An estimated pooled weighted average of RRs, using the Mantel-Haenszel fixed-effect method, with a 95% CI, was calculated. Where meta-analysis was not possible, results were presented using summary and descriptive statistics. The software used for statistical analysis was Review Manager v. 5.2.6.

## Results

### Description of studies

**Fig 1:**
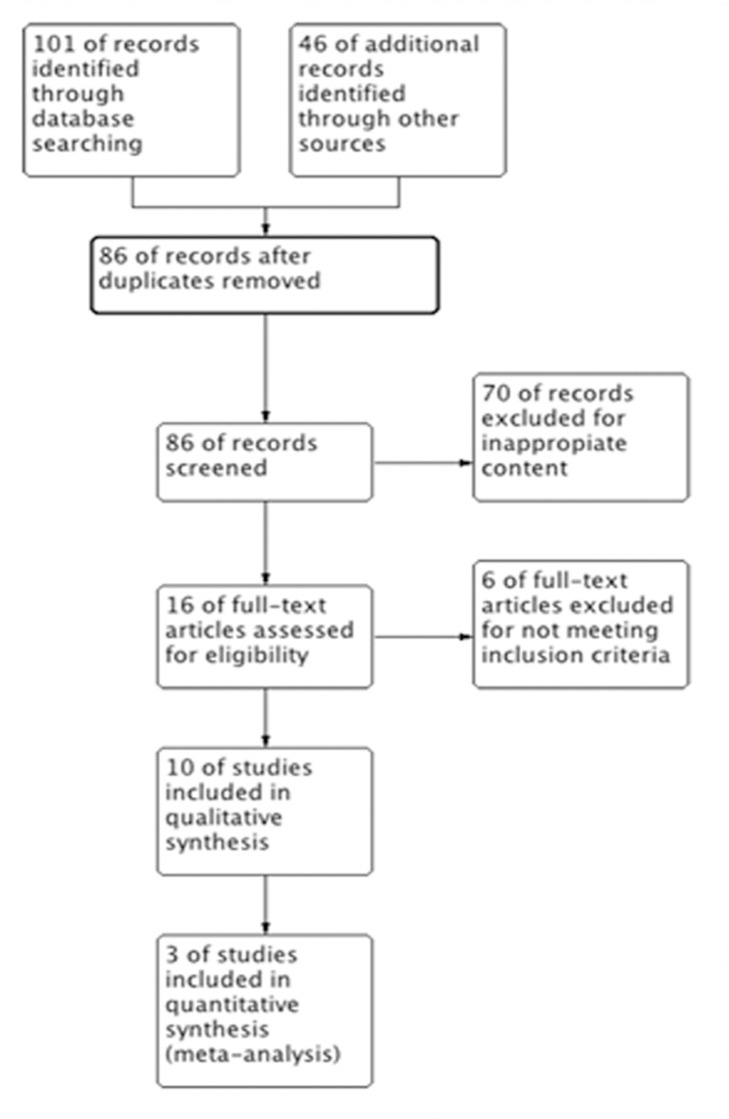
Flow chart of the study selection.

Out of n = 147 studies identified, 137 were excluded as not fulfilled inclusion criteria (Fig. 1). 10 trials were included, involving 386 subjects with medical conditions associated with CID. Among the 10 studies, five of these were set in clinics or health centers [21–25], three in public hospitals [26–28], one in the lab [29] and the rest in outpatient offices [30]. Five were based in USA [21–23, 29, 30], two in Italy [24, 27], two in France [25, 28] and one in the Netherlands [26].

All studies were structured with a study group that included OMT sessions and a control group that involved standard care or other complementary medicines procedures. Seven of the 10 studies presented information on our secondary outcome [21, 23–26, 28, 30].

Two studies explored the effect of OMT in asthmatic patients in improving asthma symptoms [21, 22]. Bockenhauer et al [21] conducted a crossover pilot study whilst Guiney et al [22] carried out an RCT.

Three studies were conducted on COPD [23, 24, 30]. Noll et al [23, 30] evaluated the baseline changes in pulmonary function parameters after the OMT intervention, while Zanotti et al [24] focused on the baseline mean changes of the 6 min walk test (6MWT) after four weeks of treatment.

As far as peripheral arterial disease (PAD) is considered, Lombardini et al conducted a case-control pilot study to investigate whether the use of OMT is effective in changing brachial artery flow-mediated vasodilation (FMV) parameters in patients with intermittent claudication [27].

Three RCTs investigated the effectiveness of visceral osteopathy in irritable bowel syndrome (IBS) patients looking at general symptoms and well-being [25, 26, 28].

Hallas et al [29] piloted a study on rats aiming at determining whether specific animal model of arthritis, used for testing the potential efficacy of anti-inflammatory agents in joint disease, produced behavioral and biomechanical changes associated with non pharmacological treatment that included osteopathic manipulative medicine and moderate exercise.

### Effect of interventions

#### Asthma

Two studies used the variation of Peak Expiratory Flow (PEF) as primary outcome [21, 22].

Bockenhauer et al [21] enrolled 10 adult smokers and non-smokers women (mean age = 47) with chronic asthma, and assigned them randomly to OMT and sham therapy. Patients acted as their own control, thus underwent both OMT intervention and sham therapy in two sessions, one week apart. OMT consisted of four techniques administered in sequential order. Sham therapy consisted of gentle manual pressure applied on different bodily regions. PEF assessments were performed 15 min before and after each intervention by two examiners. Between and within group analysis demonstrated no statistically significant differences (Tables 1 and 2).

**Table 1:** Overview of included studies of osteopathic manipulative treatment for chronic inflammatory diseases.

Author/year	Study type	Objective	Outcome measurements	Sample	Interventions	Controls
Bockenhauer 2002	Cross-over study pilot.	Evaluate the immediate effects of OMT vs sham therapy in subject with chronic asthma.	Mean changes in lower and upper thoracic excursion, and PEF differences.	10	Four recognized OMT techniques: 1) Balanced ligamentous tension in the occipitoatloid and the cervicothoracic junctions; 2) A. T. Still’s technique for “upward displacement” of the first rib; 3) Direct action release of “lower rib exhalation restriction”; 4) Diaphragmatic release.	Sham therapy
Guiney 2005	RCT	Variation of Peak Expiratory Flow after OMT in pediatric population with chronic asthma.	Mean changes in PEF rate.	90	Rib raising, muscle energy for ribs, and myofascial release.	Sham therapy
Noll 2008	RCT	Investigate the immediate effect of OMT on pulmonary function parameters in elderly subjects with chronic obstructive pulmonary disease.	Mean changes in 21 pulmonary parameters.	35	Seven standardized techniques: 1) Soft tissue; 2) Rib raising; 3) “Redoming” the Abdominal Diaphragm; 4) Suboccipital decompression; 5) Thoracic inlet myofascial release; 6) Pectoral traction; 7) Thoracic lymphatic pump with activation.	Sham therapy
Noll 2009	Observational study.	Determine the immediate effects of four osteopathic techniques on pulmonary function measures in persons with COPD relative to a minimal-touch control protocol.	Mean changes in 15 pulmonary parameters.	25	Minimal touch control and thoracic lymphatic pump with activation.	No interventions.
Zanotti 2012	RCT	Comparing the effects of the combination of pulmonary rehabilitation and OMT with pulmonary rehabilitation (PR) in patients with severely impaired COPD.	Mean change of 6MWT.	20	Osteopathic details not provided.	Sham therapy
Lombardini 2009	Case control pilot study.	Investigate the benefit of OMT, combined with lifestyle modifications and pharmacological therapy, in patient with intermittent claudication.	Mean changes in: blood tests, Brachial artery FMV, ABPI, treadmill testing, Health-related QoL.	30	Osteopathic techniques used were: 1) Myofascial release; 2) Strain/counterstrain; 3) Muscle energy; 4) Soft tissue; 5) High-velocity low- amplitude (thoracolumbar region, typically T10–L1); 6) Lymphatic pump; 7) Craniosacral manipulation.	Usual pharmacological therapy.
Hallas 1997	Laboratory study	Determine if osteopathic manipulative medicine is effective in improving behavioral and biomechanical aspects of arthritis animal models.	Baseline changes in: foot and ankle based stride length; vertical ankle and foot lift;range of motion of the ankle and knee joint.	26	Treatment consisted of passive range of motion of the right ankle and knee joint and modified muscle energy and passive myofascial stretching of the right hindlimb. Exercise in a mechanized exercise wheel.	Exercise only or no interventions.
Attali 2013	RCT	Evaluate the effectiveness of visceral osteopathy for the treatment of irritable bowel syndrome (IBS).	Qualitative evaluation of depression; constipation; diarrhea; abdominal distension; abdominal pain.	31	Global visceral technique and sacral technique were applied.	Placebo
Hundscheid 2007	RCT	Evaluate the effects of osteopathic treatment for IBS.	Change in symptoms: abdominal pain, cramps, borborygmi, diarrhea, constipation, meteorism, flatulence, feeling of incomplete evacuation of feces and presence of mucous and quality of life.	39	Black Box	Standard care
Florance 2012	RCT	Evaluate the effect of osteopathy on the severity of IBS.	Severity of IBS.	30	Osteopathic techniques administered were: direct techniques, indirect techniques, visceral techniques.	Sham therapy

OMT: osteopathic manipulative treatment; PEF: Peak Expiratory Flow; COPD: Chronic Obstructive Pulmonary Disease; PR: Pulmonary Rehabilitation; 6MWT: 6-minutes Walking Test; FMV: Flow Mediated Vasodilation; IBS: Irritable Bowel Syndrome; QoL: Quality of Life; ABPI: Ankle/Brachial Pressure Index; RCT: Randomized Controlled Trial

**Table 2:** Overview of main findings and side effects of included studies.

Author/year	Main reported findings	Side Effects
Bockenhauer 2002	Significant Increase of upper and lower thoracic excursion after OMT with a mean change respectively of 0.9 cm (SD:0.2 cm) and 0.8 cm (SD: 0.2 cm), (P = 0.005). No changes after sham procedures.	Two patients reported felling midly light headed after OMT procedure, transiently, on arising from the treatment table.
Guiney 2005	PEF increase of 4.8% in OMT group versus a mean increase of 1.4% in control group. The mean of improvement was: 13 L/minute for the OMT group, and 0 L/minute for the control group.	Data was not reported.
Noll 2008	Nonparametric ANCOVA reported statistically significant differences between the study groups pre- and post-treatment for eight of the 21 pulmonary function parameters: FEF25% L/sec (P = 0.04); FEF50%, L/sec (P = 0.008); FEF25%-75%, L/sec (P = 0.02); ERV, L (P = 0.02); RV, L (P = 0.003); TLC, L (P = 0.02); RV/TLC, % (P = 0.04); Airway resistance (cm H2O/L/s) (P = 0.04).	In the OMT group 2/18 patients reported muscle soreness, while in the sham group 4/17 subjects reported adverse effects as “elevated blood pressure in the morning”, “mild heart palpitation”, “a little muscle soreness” and “back soreness”.
Noll 2009	For the minimal-touch control protocol, only IC showed a post-treatment decrease from baseline (d = 0.57). TLP with activation had post-treatment decreases from baseline in FEFmax (d = 0.75), MVV (d = 0.59), SVC (d = 0.45), and ERV (d = 0.97); and post-treatment increases from baseline in RV (d = 0.30) and the RV/TLC ratio (d = 0.31). For TLP without activation, post-treatment FVC (d = 0.29), FEF25%-75% (d = 0.38), and MVV (d = 0.52) decreased relative to baseline and airway resistance (d = 0.30) increased relative to baseline.	1/18 subject reported side effect after minimal touch control, 4/23 after TLP with activation and 4/21 TLP without activation, rib raising produced side effects in 3/20 patients and myofascial release in 2/16 subjects.
Zanotti 2012	Within groups analysis showed that both groups reached an appreciable increase in 6MWD. In particular, the PR group gained 23.7 ± 9.7 m. Adding OMT to PR led to a further gain in 6MWD of 72.5 ± 7.5 m (p = 0.01). The difference between OMT and PR group at the end of the study was significant (48.8 m; 17–80.6 m; p = 0.04).	No adverse effects or side effects were described in either groups.
Lombardini 2009	In the control group, no changes were observed in any parameter at any time-point. In the OMT group, significant improvements were observed only after 6 months vs baseline. The 15 patients had a significant increase in ABPI, at rest and after exercise CPT and TWT were significantly longer (all p < 0.05). Brachial FMV increased significantly at months 2, 4 and 6 vs baseline. Expression of sICAM, sVCAM and IL-6 were significantly reduced at all time-points vs baseline (all p < 0.05). Questionnaire scores (physical function, role limitations due to physical problems, bodily pain and general health) overlapped in OMT patients and controls at baseline. In the OMT group they were significantly higher at month 6 (p < 0.05 vs baseline; p < 0.05 vs controls month 6).	Data was not reported.
Hallas 1997	Results demonstrate significantly improvements for each outcome parameters.	Data not available.
Attali 2013	After the intervention all symptom scores decreased in comparison to the participants’ run-in evaluation: constipation (P < 0.001), diarrhea (P = 0.003), abdominal distension (P < 0.001) and abdominal pain (P < 0.001). No significant change was observed for depressive symptoms before and after osteopathic or placebo treatment.	During the two phases of the study no side effects were reported.
Hundscheid 2007	Functional Bowel Disorder Severity Index score decreased significantly in the OMT Group as well in the standard care group, although higher in the OMT sample. Mean symptom score in the OMT group decreased from 9.1 ± 4 to 7.6 ± 4.5 at 3months, and to 6.8 ± 4 at 6 months, although not statistical significance. In the control group no change in symptom score occurred. Quality Of Life score showed an increase in the OMT group; 111 ± 22, 125 ± 20 at 3 months vs 129 ± 19 at 6 months (P < 0.009) but not in the control group.	No patients in either treatment group reported major side effects.
Florance 2012	Treatment with osteopathy significantly reduced the severity of IBS at day 7 (196±88, P < 0.01) and day 28 (224±102, P < 0.01), corresponding to a 33.7% and 25.5% improvement, respectively. The sham procedure also reduced the severity of IBS, with a 16% improvement at day 7 (244±75, P = 0.04) and an almost significant 24% improvement at day 28 (228±119, P = 0.07).	Any significant side effect was reported for both osteopathic and sham group.

OMT: Osteopathic Manipulative Treatment; SD: Standard Deviation; PEF: Peak Expiratory Flow; ANCOVA: Analysis of Covariance; FEF: Forced Expiratory Flow; RV: Residual Volume; TLC: Total Lung Capacity; IC: Inspiratory Capacity; TLP: Thoracic Lymphatic Pump; FVC: Forced Vital Capacity; MVV: Maximal Voluntary Volume; 6MWD: 6-minute walking Distance; PR: Pulmonary Rehabilitation; ABPI: Ankle/Brachial Pressure Index; CPT: Claudication Pain Time; TWT: Total Walking Time; FMV: Flow Mediated Vasodilation; sICAM: Soluble Intercellular adhesion molecule; sVCAM: Soluble Vascular Cell Adhesion Molecule; IL-6: Human Interleukin-6; IBS: Irritable Bowel Syndrome. As a secondary outcome authors measured the thoracic compliance. OMT groups significantly increased respiratory motion when compared to sham intervention groups. The mean change in upper and lower thoracic excursion was statistically significant between groups (Tables 1 and 2).

Guiney et al [22] enrolled 140 asthmatic children (range 5–17 y) and randomly assigned to OMT group and sham control group. The main outcome was the baseline variation of PEF before and after treatment. Results demonstrated that OMT group significantly increase PEF rates compared to control (PEF_OMT_: 13.0 (27.4); PEF_sham_: 0.3 (35.5)). Within group analysis demonstrated that the OMT group moved from 7 L to 19 L/minute, whilst the control group did not change (10 L/minute) (Tables 1 and 2).

#### Chronic obstructive pulmonary disease (COPD)

Three studies addressed the effectiveness of OMT on COPD [23, 24, 30].

Noll et al. [30] enrolled 35 elderly patients (mean age = 72) with primary diagnosis of COPD hypothesizing that a single multi-technique OMT session would produce an immediate effect on pulmonary function parameters compared to light-touch sham control treatment. A nonparametric ANCOVA showed statistically significant mean differences between groups in eight of the 21 pulmonary function parameters analyzed (Tables 1 and 2).

The day after the subjects received the treatment, a telephone survey was conducted with the aim at assessing the success of blinding, subjective perception of the intervention received and adverse effects (Tables 1 and 2).

Another piece of research from Noll and colleagues [23] was conducted looking at the immediate effect of four single-technique treatment sessions on a sample of 25 COPD patients (mean age = 68; SD = 8). This RCT, comparing four OMT groups to a minimal touch control, showed different results according to the type of technique used (Table 2). Overall, the use of any single osteopathic technique was associated with a moderate post treatment decrease of pulmonary function.

Zanotti et al. [24] included 20 patients with severe COPD aged 64 (SD = 5). The primary outcome measure of their study was the mean change of 6MWT in people treated with four OMT sessions plus pulmonary rehabilitation (PR) compared with people treated with PR only over a period of one month. Secondary outcomes were baseline changes in pulmonary parameters.

Results demonstrated that combining OMT with PR produced an additional gain in 6MWT of 49m (95% CI 17 to 81m). In addition, OMT plus PR led to a significant improvement of the residual volume compared to control group (Tables 1 and 2).

**Fig 2:**
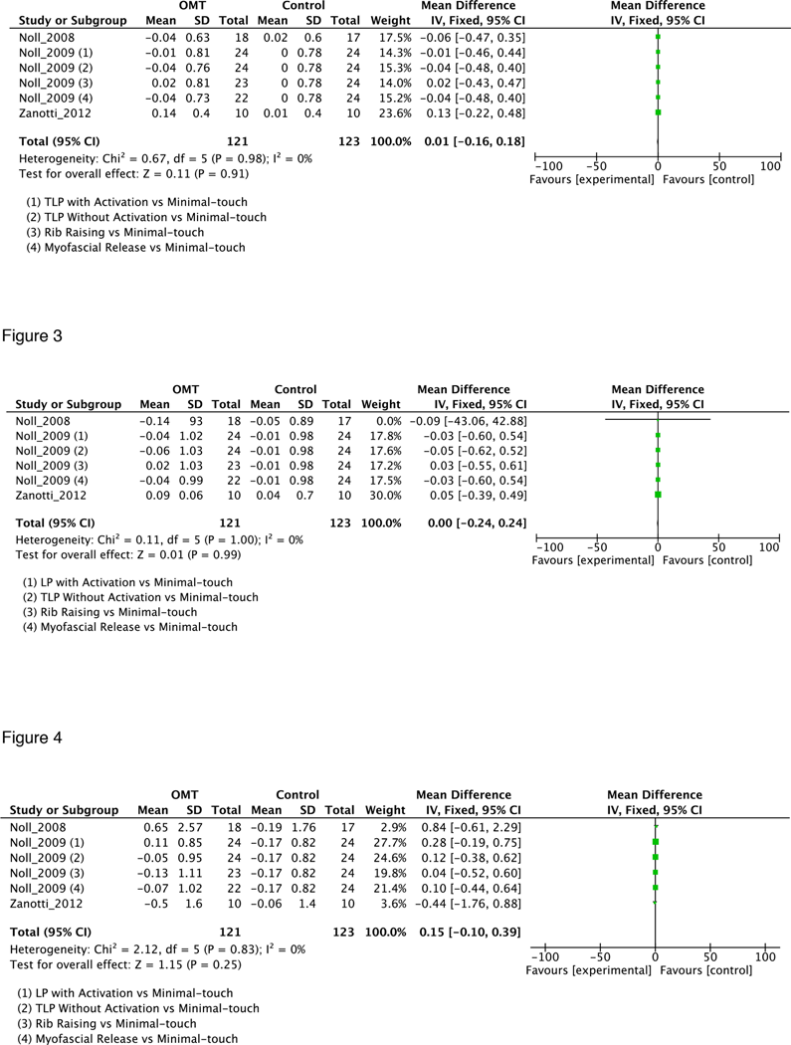
Forest plot of comparisons, OMT for COPD parameters. Outcomes: A, forced expiratory volume in the first second (FEV1); B, forced vital capacity (FVC) and C, residual volume (RV). CI, confidence interval; SD, standard deviation.

A meta-analysis was conducted on these three studies [23, 24, 30] with the aim to evaluated the efficacy of the type of osteopathic techniques on three pulmonary parameters: forced expiratory volume in the first second (FEV1), forced vital capacity (FVC) and residual volume (RV). Results showed no statistically significant differences between techniques on FEV1, FVC and RV parameters (Fig. 2).

#### Peripheral arterial disease (PAD)

The only study published on PAD was conducted by Lombardini et al [27]. Authors investigated whether 6-months osteopathic treatments, in combination with lifestyle modifications and pharmacological therapy, could improve endothelial function and functional performances in a sample of 30 adult male patients (mean age = 69; SD = 8) with PAD and intermittent claudication.

Within group analysis showed significant changes only in the OMT group when compared end-of-study and baseline endothelial and functional values (Tables 1 and 2). Moreover, OMT group significantly improved health scores (p < 0.05).

#### Irritable bowel syndrome (IBS)

Three studies were carried out enrolling patients with IBS [25, 26, 28] (Tables 1 and 2).

Hundscheid et al [26] conducted a RCT on 36 patients (mean age = 43) with IBS investigating the effect of five osteopathic sessions compared with standard care in changing symptoms and quality of life. The study used the standardized IBSQOL 2000 questionnaire and the Functional Bowel Disorder Severity Index as outcome tools. After six months, results showed a significant decrease of Functional Bowel Disorder Severity Index in the OMT group compared with the standard care group. Furthermore the IBSQOL score increased significantly in the OMT group compared to the standard care group.

Florance et al [28] conducted a RCT enrolling 30 subjects affected by IBS (mean age = 48, SD = 17). Results showed that osteopathic treatment improved quality of life and reduced the severity of IBS symptoms by 33% after 7 days and 25% after 28 days. Sham procedure reduced the severity of IBS by 16% at day 7 and 24% at day 28. Secondary outcomes concerning psychological aspects (depression and fatigue) did not show any significant difference at any time point between groups. Level of satisfaction was higher in the OMT group compared to control.

Attali et al [25] included 31 patients with refractory IBS aged 50 (SD = 2) and randomized them in a three sessions of visceral osteopathic manipulation and three sessions of sham therapy. Outcomes were baseline changes in constipation, diarrhea, abdominal distention, abdominal pain and depression. After 10 weeks, authors claimed that visceral osteopathic manipulation produced an amelioration of symptoms associated with IBS and a reduction of rectal hypersensitivity. No significant results were reported for colonic transit time.

#### Arthritis

Considering arthritis, the only research carried out was performed by Hallas et al [29]. Authors created a unilateral arthritis rat model and assigned 18 animals to treated (OMT) and untreated groups. A healthy group (n = 8) was included as control. The primary outcome of the study was to determine whether non pharmacological treatment (OMT + exercise) could produce behavioral and biomechanical changes in treated subjects compared with healthy controls (untreated group).

Results showed significant increase in foot-based stride length at the end of the experiment in the treated group versus the untreated group which showed no changes in the same parameter over time (Tables 1 and 2). Furthermore, in the ankle-based stride length assessment, the OMT group increased the stride length over the untreated group but with a non significant difference. The OMT group also showed significantly greater improvement in vertical ankle lift over time compared to the untreated arthritic group (Table 1).

#### Side Effects

Only seven studies reported data on side effects [21, 23–26, 28, 30]. In five studies [21, 24–26, 28] none of the participants showed side effects after osteopathic treatments (Table 2). In the study conducted by Noll et al. [23], 14 subjects over 25 reported mild side effects characterized by musculoskeletal soreness or pain. Post-hoc calculation of RR showed a reduction of side effects in the minimal touch control group compared to all other groups (data not showed). A further study conducted by Noll et al [30] reported two patients with symptoms of muscle soreness after the OMT session, while in the sham group the side effects were recorded in four subjects who reported “elevated blood pressure in the morning”,”mild heart palpitations”, “a little muscle soreness” and “back was a little sore”. Again, post-hoc RR computations demonstrated no significant reduction of side effects in the study group compared to controls (data not showed).

#### Interventions

Considering osteopathic treatment and control procedures, high heterogeneity was revealed across studies in terms of: type of techniques used, length of the session, dose and duration of manipulations, practitioners’ background, settings for interventions and type of control group.

Bockenhauer et al [21] utilized pre-determined manual protocols based on four OMT techniques applied in the following sequential order: balanced ligamentous tension in the occipito-atloid and the cervicothoracic junctions; Still’s technique for “upward displacement” of the first rib; direct action release of “lower rib exhalation” restriction, and diaphragmatic release. The entire intervention last 10 to 15 minutes. The distance between treatments was at least 1 week. Sham procedures took place in the same room, with the subjects in the same position of the OMT session. Sham techniques in this study consisted in gentle manual pressure to the region of the thoracic outlet, occipito-atloid and cervicothoracic junctions and epigastric region. The upper extremities were circumducted at the shoulder through a partial range of passive motion. No details on settings and practitioners who carried out treatments were provided.

Guiney et al [22] used three pre-determined osteopathic manipulative techniques administered in random sequence: rib raising, muscle energy for ribs and myofascial release. An osteopathic physician performed all osteopathic sessions. Number of treatments, dose, length and period of treatment were not reported. An allopathic physician performed the sham therapy using soft touch on different bodily regions such as rib cage, paraspinal muscles and abdominal diaphragm areas mimicking the osteopathic techniques without applying any type of therapeutic process. No details on settings were provided.

Attali et al [25] performed a pre-determined standardized approach consisting of one general visceral technique in association with local techniques on hypersensitive areas of the body and one sacral technique. OMT group received 3 sessions, separated by two weeks interval, lasting 45 minutes each. As far as the placebo treatment is considered, the practitioner performed a superficial abdominal massage in the same areas of the OMT procedure, without any internal organ mobilization. Number of treatments and length of sessions overlapped the OMT criteria. All the manipulations were performed by a single senior osteopath. No details on settings were reported.

Hundscheid et al [26] used a patient need-based approach applying five osteopathic sessions one every 2–3 weeks. One osteopath was in charge of performing OMT. The standard care group received a diet rich in fiber. Moreover, extra fiber and laxatives could have been administered to the standard care group in case of constipation and loperamide was used if necessary in case of predominant diarrhea. In case of abdominal cramps, mebeverine was prescribed. No sham osteopathic treatment was applied. The osteopathic sessions were performed in a private osteopathic practice whilst standard care was administered in a public hospital. No details on length of each session was provided.

Florance et al [28] used a standardized osteopathic procedure consisting in three techniques applied in sequential order: one direct technique where the osteopath applied manual pressures on each segment of the spine for 90 seconds, followed by one indirect technique where the osteopath manipulated the spine segment in all directions and one final visceral technique to relieve imbalances between the motion of all the organs. Patients received two sessions of manipulative treatment at a 7-day interval. Each session lasted 60 min. One single osteopath administered OMT. The sham procedure consisted of a gentle massage in the same areas treated in the OMT sessions. No information on settings was provided.

Lombardini et al [27] planned eight sessions in six months with a session treatment time of approximately 30 minutes. Sessions were organized as follows: every two weeks during the first two months, every three weeks during the forth, fifth and sixth months. A wash-out period of one month was scheduled between month two and three. The OMT sessions were performed by an osteopath while physicians and nurses collected blood samples and assessed vascular parameters for the control group. The study was conducted in two adjacent rooms of a public hospital unit. No details on osteopathic approach were reported.

Zanotti et al [24] used a patient need-based approach programming one treatment per week for four weeks. Each session lasted 45 min. The examination procedures were conducted by an osteopathic practitioner with emphasis on neuromusculoskletal system. Information on soft manipulation procedure, control group and setting of the interventions were not provided.

Noll et al [23] applied five single standardized treatment protocols in random order. Techniques used were: minimal touch control, thoracic lymphatic pump with and without activation, rib raising and myofascial release. Between each treatment protocol session, a 4-week washout period was planned. The duration of each treatment protocol ranged from five to 10 minutes depending on the type of technique. The osteopathic treatments were administered in an outpatient office by two osteopathic physicians. The pulmonary function tests were conducted by certified respiratory therapists.

The other research programme conducted by Noll et al [30] administered seven standardized osteopathic manipulative techniques: soft tissue, rib raising, indirect myofascial release, sub-occipital decompression, thoracic inlet myofascial release, pectoral traction, thoracic lymphatic pump with activation. The treatment session duration was 20 minutes. Sham manipulations mimicked in terms of dose, length and duration the OMT session. An outpatient office setting was chosen for the study. No other details were reported.

Hallas et al [29] dealt with rats and utilized a sequence of pre-determined techniques consisting of passive range of motion of the right ankle and knee joint, modified muscle energy techniques and passive myofascial stretch. Techniques were applied 10 times a day for five days a week for six weeks by student physicians with at least one year of training in osteopathic manipulative medicine. The duration of each technique was 10 seconds. The control group performed sessions consisting of five minutes of moderate exercise on a mechanical wheel. Dose and total duration of treatment was consistent with the OMT group. All sessions were performed in the laboratory.

### Risk of bias in included studies

#### Allocation

**Fig 3:**
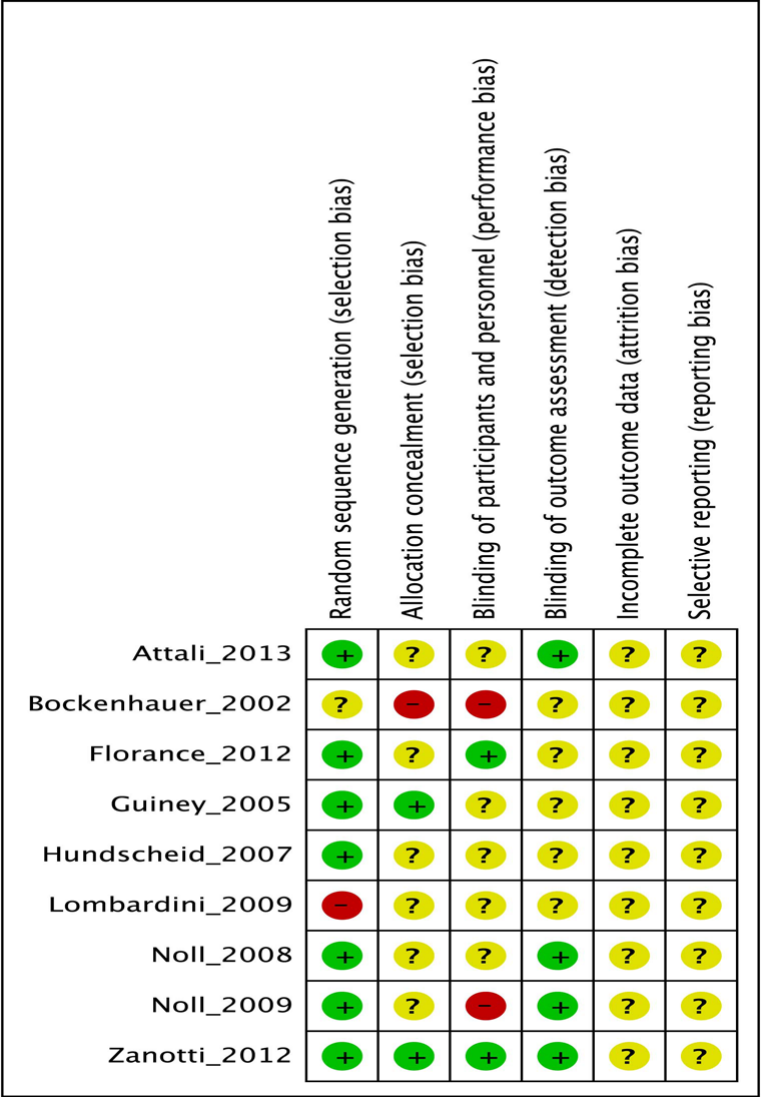
Risk of bias for included studies. +, low risk of bias;-, high risk of bias,?, unclear risk of bias.

Out of 10 studies included, six were RCTs [22, 24–26, 28, 30] but only two employed an adequate randomization method: Attali et al [25] used random permutations generated by a computer software, while Zanotti et al [24] drawn a randomization based on computer-generated list of random numbers within the range of 1 to 20. The remaining four did not specify the randomization method used [22, 26, 28, 30] (Fig. 3).

Two research used a 2:1 allocation ratio [22, 28]. Hundscheid et al [26] allocated patients using closed envelopes. Patients included by Noll et al [30] were randomly assigned to either the OMT or sham protocol group using stratification by the severity of airflow obstruction (Fig. 3).

The remaining four non-RCT studies were designed as observational [23], pilot cross-over [21], laboratory [29] and case control [27], exposed, therefore, to allocation biases (Fig. 3).

#### Blinding of participants and personnel

The majority of the studies did not report data on blinding of participants and personnel producing an unclear risk of performing bias [21, 22, 25–27, 29, 30].

One study had a high risk of bias as unblinding of osteopathic physicians and patients [23].

Low risk of bias was revealed for Florance et al [28] as the osteopath was not aware of the clinical situation and was not involved in data analysis. Similarly Zanotti et al [24] claimed that patients remained blinded to the randomization and were not able to determine the study arm allocation (Fig. 3).

#### Blinding of outcome assessors

Five studies reported an acceptable blinding of the outcome assessors as all measurements were performed by a clinician or specialized therapists, blinded to the treatment applied [21, 23–25, 30].

All the other five included studies did not report any information [22, 26–29] (Fig. 3).

#### Selective bias

Study protocols were not available and although requested, the assessment was not possible to carry out (Fig. 3).

#### Other bias

The quality of studies included was further assessed considering other reported information regarding: conflict of interest, reporting funding source, ethical approval, informed consent, confidentiality, declaration of interests, access to data, trial registration, data collection, data management and data monitoring committee. Taking into account conflict and declaration of interests only five studies specified appropriate information [23–25, 28, 30]. Regarding source of funding, only Noll et al [23, 30] and Hallas et al [29] reported complete details on grants received. All studies included apart from Hallas et al [29] declared ethical statement and informed consent approval. None of the research detailed any information regarding confidentiality, access to data, trial registration, data collection, data management and data monitoring committee (S1 Table).

## Discussion

The present systematic review aimed at evaluating the effectiveness of omt in patients with pathologies classified also as cid. It included 10 research studies (one laboratory study [29], one cross-over pilot study [21], one observational [23], one case control pilot study [27], six RCTs [22, 24–26, 28, 30]) and 386 patients. Overall, results suggest a potential effect of osteopathic medicine in medical conditions potentially associated with CID compared to no treatment or sham therapy although data remains elusive. Different pathologies were addressed, in particular asthma, COPD, PAD, IBS and arthritis. Considering asthma two studies [21, 22] were included and reported contradictory results over the same outcome but on different population. Bockenhauer et al [21] reported no effect on baseline changes of PEF in a small sample of adults, while Guiney et al [22] showed a positive effect of OMT in children. Out of three studies [23, 24, 30] dealing with COPD, the meta-analysis showed no statistical effect of OMT on FEV1, FVC and RV, although positive effects were reported by each study. PAD case control study [27] demonstrated an amelioration in endothelial functional values on the OMT group only. RCTs carried out on IBS patients [25, 26, 28] demonstrated a statistical significant difference in functional and subjective parameters in favor of OMT. Finally, Hallas et al [29] dealt with rats arthritis and showed positive results in terms of joint mobility.

Included studies were conducted in both clinical and private practice settings, and in different European countries as well as in the United States, which would suggest that the findings would be applicable in these contexts. Studies also included treatments carried out by both interns and by experienced practitioners, with no apparent impact on outcomes.

Included studies had several limitations which should be considered when interpreting these findings. The characteristics of participants were poorly reported in most of the studies. Additionally, there was a paucity of information about the setting of osteopathic care, the nature of intervention and the content of the training provided to the staff. Although this systematic review was focused on the effectiveness of OMT, we noticed the general lack of data on integration of other aspects of the health system (e.g. health information systems, leadership, financing etc.). The studies were mostly based on single health facilities and given high levels of short-term outcomes. Many studies did not report quantitative data on the proportion of patients having adverse events.

Considering the quality of evidence, more than one article showed methodological limitations. Among RCTs the majority of studies did not fully report details to assess the risk of bias. Numerous trials appear to deviate from international guidelines for reporting clinical trials, leading to difficulties in assessing the quality of each research study. Similar patterns were shown in observational studies. Recent literature has shown common concerns across quality of evidence evaluation and reporting for OMT. Turner et al. pointed out that the quality of RCTs reported is inadequate [31] while Hopewell et al. documented several failures in peer-review processes in detecting significant limitations across published RCTs [32].

Furthermore, out of 10 studies included, only 6 [21, 22, 24, 25, 28, 30] employed sham therapy as control, the others used standard therapies or did not use any control. Heterogeneity between trials was revealed for study samples and osteopathic care. The latter varied from pre-determined protocol to black-box method using different techniques from balanced ligamentous tension, Still techniques, muscle energy, myofascial release to cranio-sacral approach. However, similar techniques were reported in several studies [23, 24, 27, 30] which used thoracic lymphatic pump doming diaphragm and myofascial release techniques, but with different methods of interventions. As sample size was limited in each study, results were summarized based on reported frequencies. This yielded higher sample size and higher external validity on cost of biases introduced by the heterogeneity of the studies and their different sample sizes.

Heterogeneity was also showed for the frequency of OMT session, settings and outcome measures. It should be acknowledged that complete blinding for manual treatments is inherently impossible or difficult to achieve. Therefore, these clinical and methodological heterogeneities prevented consistent reporting of data. Furthermore, the overall risk of adverse events was not possible to quantify as no consistent data were provided.

To the best of our knowledge this is the first review dealing with chronic inflammatory disease in the field of osteopathy. The main potential cause of bias in the review process was minimized by ensuring that the decisions regarding eligibility for inclusion and data extraction were completed independently by two review authors, disagreements between whom were resolved by discussion and consensus.

A possible limitation of the present systematic review is publication bias, of which there are some potential sources [33]. No attempt was made to identify unpublished research, which is more likely to have negative outcomes [34, 35]. Nevertheless, the efforts to retrieve unpublished data from trials are also expected to be biased [34]. The search strategy may have left out relevant studies not currently indexed, but by including citation tracking of non-indexed journals omissions should have been reduced at a minimum. Optimally, reviews should include all trials regardless of language [36–38]. Although an attempt was made to identify trials in all languages, the chance that some important studies may have been not reported have to be recognized. Additionally, the ability to detect statistically significant difference in subgroup analyses was limited by the low number of studies reporting on coverage of individual interventions. Eventually, trials selected for this review included patients with pathologies associated with CID and not as primary diagnosis of CID. This is due to the intrinsic limitations of CID as a general classifications of diseases, which considers a list of different diseases with common pathogenesis but different signs and symptoms.

## Conclusion

The present systematic review showed inconsistent data on the effect of OMT in the treatment of pathologies associated with CID. The majority of the studies are generally small and methodologically prone to bias affecting, therefore, the generalisability of findings. Moreover, very little research has been conducted preventing any possible additional speculation on the effectiveness of OMT. Researchers should be aware of methodological and clinical limitations of the current osteopathic literature on CID and propose more robust and rigorous RCTs to clarify many unsolved questions regarding the effectiveness of OMT on medical conditions also classified as CID. Economic evaluation of any benefits would also be needed to inform policymakers, stakeholders and the guidance provided to and by physicians.

## Supporting Information

S1 Checklist The PRISMA Checklist. (PDF)

S1 Table Risk of ‘other’ bias for included studies. Abbreviations: HR High risk of bias, LR Low risk of bias, UC Unclear.
(PDF)
