# Supplementary material for: Chronic Inflammatory Disease and Osteopathy: A Systematic Review
Source: PLoS One. 2015 Mar 17;10(3):e0121327. doi: 10.1371/journal.pone.0121327 (PMC4363664; doi:10.1371/journal.pone.0121327)
Supplement: S1 Table — Abbreviations: HR High risk of bias, LR Low risk of bias, UC Unclear. (PDF) [file pone.0121327.s002.pdf]

Author/year	Conflict of interest	Reporting funding source	Ethical approval	Informed consent	Confidentiality	Declaration of interest	Access to data	Trial registration	Data collection	Data management	Data monitoring committee
Bockenhauer 2002	HR	HR	LR	LR	HR	HR	HR	HR	HR	HR	HR
Guiney 2005	HR	HR	LR	LR	HR	HR	HR	HR	HR	HR	HR
Noll 2008	LR	LR	LR	LR	HR	LR	HR	HR	HR	HR	HR
Noll 2009	LR	LR	LR	LR	HR	LR	HR	HR	HR	HR	HR
Zanotti 2012	LR	HR	LR	LR	HR	LR	HR	HR	HR	HR	HR
Lombardini 2009	HR	HR	LR	LR	HR	HR	HR	HR	HR	HR	HR
Hallas 1997	HR	LR	HR	NA	NA	HR	NA	NA	NA	HR	HR
Attali 2013	LR	HR	LR	LR	HR	LR	HR	HR	HR	HR	HR
Hundscheid 2007	HR	HR	LR	LR	HR	HR	HR	HR	HR	HR	HR
Florance 2012	LR	HR	LR	LR	HR	LR	HR	HR	HR	HR	HR
